# JAK Inhibitors and Modulation of B Cell Immune Responses in Rheumatoid Arthritis

**DOI:** 10.3389/fmed.2020.607725

**Published:** 2021-02-05

**Authors:** Rita A. Moura, João Eurico Fonseca

**Affiliations:** ^1^Instituto de Medicina Molecular João Lobo Antunes, Faculdade de Medicina, Universidade de Lisboa, Lisbon, Portugal; ^2^Rheumatology Department, Hospital de Santa Maria, Centro Hospitalar Universitário Lisboa Norte (CHULN), Lisbon Academic Medical Centre, Lisbon, Portugal

**Keywords:** JAK-STAT pathway, JAK inhibitors, B cells, cytokines, rheumatoid arthritis

## Abstract

Rheumatoid arthritis (RA) is a chronic, systemic immune-mediated inflammatory disease that can lead to joint destruction, functional disability and substantial comorbidity due to the involvement of multiple organs and systems. B cells have several important roles in RA pathogenesis, namely through autoantibody production, antigen presentation, T cell activation, cytokine release and ectopic lymphoid neogenesis. The success of B cell depletion therapy with rituximab, a monoclonal antibody directed against CD20 expressed by B cells, has further supported B cell intervention in RA development. Despite the efficacy of synthetic and biologic disease modifying anti-rheumatic drugs (DMARDs) in the treatment of RA, few patients reach sustained remission and refractory disease is a concern that needs critical evaluation and close monitoring. Janus kinase (JAK) inhibitors or JAKi are a new class of oral medications recently approved for the treatment of RA. JAK inhibitors suppress the activity of one or more of the JAK family of tyrosine kinases, thus interfering with the JAK-Signal Transducer and Activator of Transcription (STAT) signaling pathway. To date, there are five JAK inhibitors (tofacitinib, baricitinib, upadacitinib, peficitinib and filgotinib) approved in the USA, Europe and/ or Japan for RA treatment. Evidence from the literature indicates that JAK inhibitors interfere with B cell functions. In this review, the main results obtained in clinical trials, pharmacokinetic, *in vitro* and *in vivo* studies concerning the effects of JAK inhibitors on B cell immune responses in RA are summarized.

## Introduction

The success of B cell depletion therapy with rituximab in autoimmune diseases such as rheumatoid arthritis (RA) has reinforced the important role that B cells have in the development of these conditions (1, 2). Indeed, B cells can be responsible for autoantibody production, antigen presentation and T cell activation and/ or cytokine and chemokine release that contribute to disease pathogenesis (3). RA is a chronic, systemic immune-mediated disease that mainly affects the small joints of hands and wrists and, though often ameliorated by treatment, can lead to bone and cartilage destruction (4, 5). Treatment options in RA include non-steroid anti-inflammatory drugs (NSAIDs), corticosteroids, synthetic and/or biologic disease modifying anti-rheumatic drugs (DMARDs). Nevertheless, despite the progresses achieved in the last decades in RA pharmacotherapy, few patients reach sustained remission and refractory disease remains a significant challenge (6–8). Janus kinase (JAK) inhibitors or JAKi are recently approved oral medications with therapeutic application in myeloproliferative disorders and inflammatory diseases such as RA. JAKi function by inhibiting the activity of one or more of the JAK family of enzymes [JAK1, JAK2, JAK3, and tyrosine kinase 2 (TYK2)], thus interfering with the JAK-Signal Transducer and Activator of Transcription (STAT) signaling pathway (9, 10). There are currently five JAK inhibitors (tofacitinib, baricitinib, upadacitinib, peficitinib, and filgotinib) approved in the USA, Europe and/ or Japan for RA treatment. Furthermore, an additional JAKi (decernotinib) is under investigation for RA treatment in clinical trials (11, 12). Although the number of studies exploring the effect of JAK inhibitors on B cells in the context of RA is limited, evidence from the literature indicates that JAKi also interfere with B cell functions. In this review, we summarize the main results obtained so far in clinical trials, pharmacokinetic, *in vitro* and *in vivo* studies concerning the effects of JAK inhibitors on B cell immune responses in RA.

## B cells and Rheumatoid Arthritis

B cells play several important roles in the development of RA (13). B cells produce autoantibodies, such as rheumatoid factor (RF) and anti-citrullinated protein antibodies (ACPA), which form immune complexes that deposit in the joints and contribute to the inflammatory process through complement and cellular activation. Furthermore, B cells act as efficient antigen presenting cells (APC) that activate T cells through the expression of costimulatory molecules. B cells also secrete cytokines and/ or chemokines that promote leukocyte infiltration in the joints and the development of ectopic lymphoid structures, thus aggravating angiogenesis, pannus formation and synovial hyperplasia. In addition, the therapeutic efficacy of rituximab, an anti-CD20 monoclonal antibody that specifically depletes B cells, in RA patients has unequivocally supported B cell targeted therapies in RA pathogenesis (1, 2, 14). Of note, previous studies by our group have demonstrated that untreated very early RA patients (with <6 weeks of disease duration) have alterations in circulating memory B cell subpopulations (15); a cytokine profile that supports an early B cell activation (16, 17); and changes in B cell gene expression levels relevant for B cell maturation and differentiation (18). These data reinforce an active role of B cells in RA pathogenesis from early disease onset. Moreover, we have recently shown that in RA, treatment with tumor necrosis factor (TNF)-inhibitors and the interleukin (IL)-6 receptor (IL-6R) antagonist tocilizumab affect B cell phenotype and IgD-CD27- memory B cells in peripheral blood (19). Importantly, clinical relapse observed in B cell depleted RA patients has been associated with B cell repopulation (20–22). In fact, the results observed in RA patients following B cell depletion therapy with rituximab suggest that alterations in the expression of B cell activating factor (BAFF)-binding receptors and an increase in class-switch recombination process, particularly in memory B cell subsets, might be associated with the re-establishment of active disease (23). Interestingly, it has also been recently demonstrated for the first time that the autoantibodies commonly found in RA patients, RF and ACPA, express the inherently autoreactive 9G4 idiotope, thus supporting an activation of autoreactive 9G4+ B cells in RA (24). Additionally, it has been recently suggested that the pattern of B cell distribution in synovial tissue from untreated early RA patients can be associated to a specific pathotype classification with cellular and molecular synovial signatures that might help to predict disease severity, radiographic progression and therapeutic response (25, 26).

## Cytokines as Key Players in Rheumatoid Arthritis Pathogenesis

**Figure 1:**
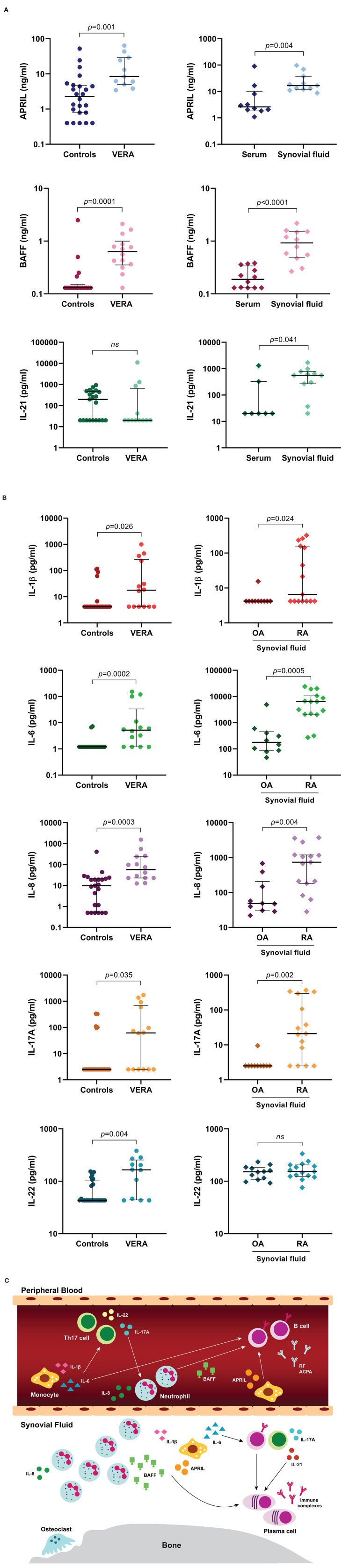
Cytokine profile present in peripheral blood from very early rheumatoid arthritis (VERA) patients and synovial fluid from established RA. A group of cytokines directly related with B cell activation, differentiation and survival was quantified in serum samples from untreated very early rheumatoid arthritis (VERA) patients with <6 weeks of disease duration when compared to healthy controls **(A)**. In addition, serum and synovial fluid samples from established treated RA patients were also analyzed for comparison **(A)**. Cytokines related with neutrophil and Th17 cells activation were also quantified in serum samples from VERA patients and healthy individuals **(B)**. Furthermore, synovial fluid from established treated RA and osteoarthritis (OA) patients was analyzed for comparison **(B)**. Statistical analysis of data was performed with GraphPad Prism (GraphPad Software, San Diego, CA, USA). Lines in graphs represent median values with interquartile range. Non-parametric Mann-Whitney test was used for comparisons between two independent groups. Differences were considered statistically significant for *p* < 0.05. Data represented in Figures 1A,B were adapted from previous published studies by our group (16–18), according to the terms of the Creative Commons license (http://creativecommons.org/licenses/by/4.0/). Figure 1C is an illustration representative of the cytokine profile present in peripheral blood from VERA patients and synovial fluid from established RA supported by previous published studies by our group (16–18). To sum up, RA patients have a cytokine profile in peripheral blood that favors B cells, neutrophils and Th17 cells activation since the first weeks of disease development. In a chronic phase of the disease, the cytokine pattern present locally in the joints supports the intervention of activated monocytes, neutrophils, T and B cells and plasma cell differentiation **(C)**. ACPA, anti-citrullinated protein antibodies; APRIL, a proliferation-inducing ligand; BAFF, B cell activating factor; IL, interleukin; *ns*, non-significant; OA, osteoarthritis; RA, rheumatoid arthritis; RF, rheumatoid factor; Th17, T helper 17; VERA, very early rheumatoid arthritis.

Cytokines are a large family of secreted proteins that play important roles in the immune system, namely in cell differentiation, maturation and signaling. Cytokines can be produced by several types of immune cells, including macrophages, B cells, T cells and mast cells, as well as endothelial cells, fibroblasts and various stromal cells. Of note, cytokines can be major drivers of autoimmunity and inflammation. In RA, several cellular interactions and complex cytokine networks occur that contribute to disease pathogenesis (13). In fact, it has been demonstrated that cytokines including IL-1 beta (IL-1β), IL-2, IL-3, IL-6, IL-7, IL-8, IL-12, IL-15, IL-17, IL-18, IL-19, IL-20, IL-21, IL-23, IL-32, IL-33, IL-35, TNF, interferon-alpha/gamma (IFN-α/γ) and granulocyte-macrophage colony-stimulating factor (GM-CSF) have important roles in RA physiopathology as they contribute to the induction and maintenance of inflammation (13, 27–30). The inflammatory process that develops in RA leads to a cellular infiltration of the synovial membrane, angiogenesis, pannus formation, swelling, and pain. The interactions between B and T cells result in the activation and differentiation of plasma cells, which are responsible for the production of autoantibodies (RF, ACPA). These autoantibodies form immune complexes that can activate complement and stimulate cells such as monocytes by binding to their Fc-gamma receptors (FcγR), triggering cytokine and/ or chemokine release that cause inflammation. Indeed, activated monocytes, neutrophils, and fibroblasts can release high levels of cytokines such as IL-1, IL-6, and TNF, that further activate not only B and T cells, but also chondrocytes and osteoclasts, thus contributing to cartilage and bone destruction (13). Furthermore, cytokines directly related with B cell activation and survival such as A proliferation-inducing ligand (APRIL) and BAFF (31–35), which can be produced by activated monocytes and neutrophils, have been shown to contribute to RA development from an early phase in disease onset (17). Moreover, increased serum levels of BAFF have been suggested to have an important role in B cell triggering during clinical relapse after B cell depletion therapy (23). Previous studies developed by our group have demonstrated that untreated very early RA (VERA) patients (with <6 weeks of disease duration) have a cytokine pattern in circulation that supports an early activation of not only B cells, but also neutrophils and Th17 cells (16, 17) (Figure 1). Indeed, we have found that VERA patients have higher serum levels of APRIL and BAFF when compared to other very early arthritis (non-RA) patients, established RA and healthy controls (17). We also observed that established RA patients have significantly increased synovial fluid levels of APRIL, BAFF and IL-21, a cytokine important for plasma cell differentiation (17) (Figure 1A). Additionally, we found that VERA patients have increased serum levels of cytokines that promote neutrophil recruitment and activation (IL-8), Th17 cells polarization (IL-1β and IL-6) and Th17 cells-derived cytokines (IL-17A and IL-22) (16) (Figure 1B). Also, the elevated IL-1β, IL-6, IL-8, and IL-17A levels observed in the synovial fluid of established RA patients support a local role for these cytokines in synovial inflammation and bone erosion (16) (Figures 1B,C). In fact, IL-17 has been shown to induce osteoclastogenesis, thus contributing for bone resorption (36, 37). Moreover, IL-6 can support the activation and recruitment of autoreactive B cells toward RA synovium (38, 39), leading to an exacerbation of inflammation through autoantibody production and immune complex deposition (40, 41) (Figure 1C). Of note, treatment of VERA patients with corticosteroids and methotrexate (MTX), although effective in clinical improvement had no impact on the cytokine pattern in circulation (16, 17). Importantly, the success of biological therapies that directly target key cytokines such as TNF inhibitors (adalimumab, infliximab, etanercept, golimumab and certolizumab); tocilizumab (an IL-6R antagonist) and anakinra (an IL-1R antagonist) in RA further reinforce the relevance of these small proteins in disease development (42–46).

## JAK-STAT Signaling Pathway in Health and Disease

**Figure 2:**
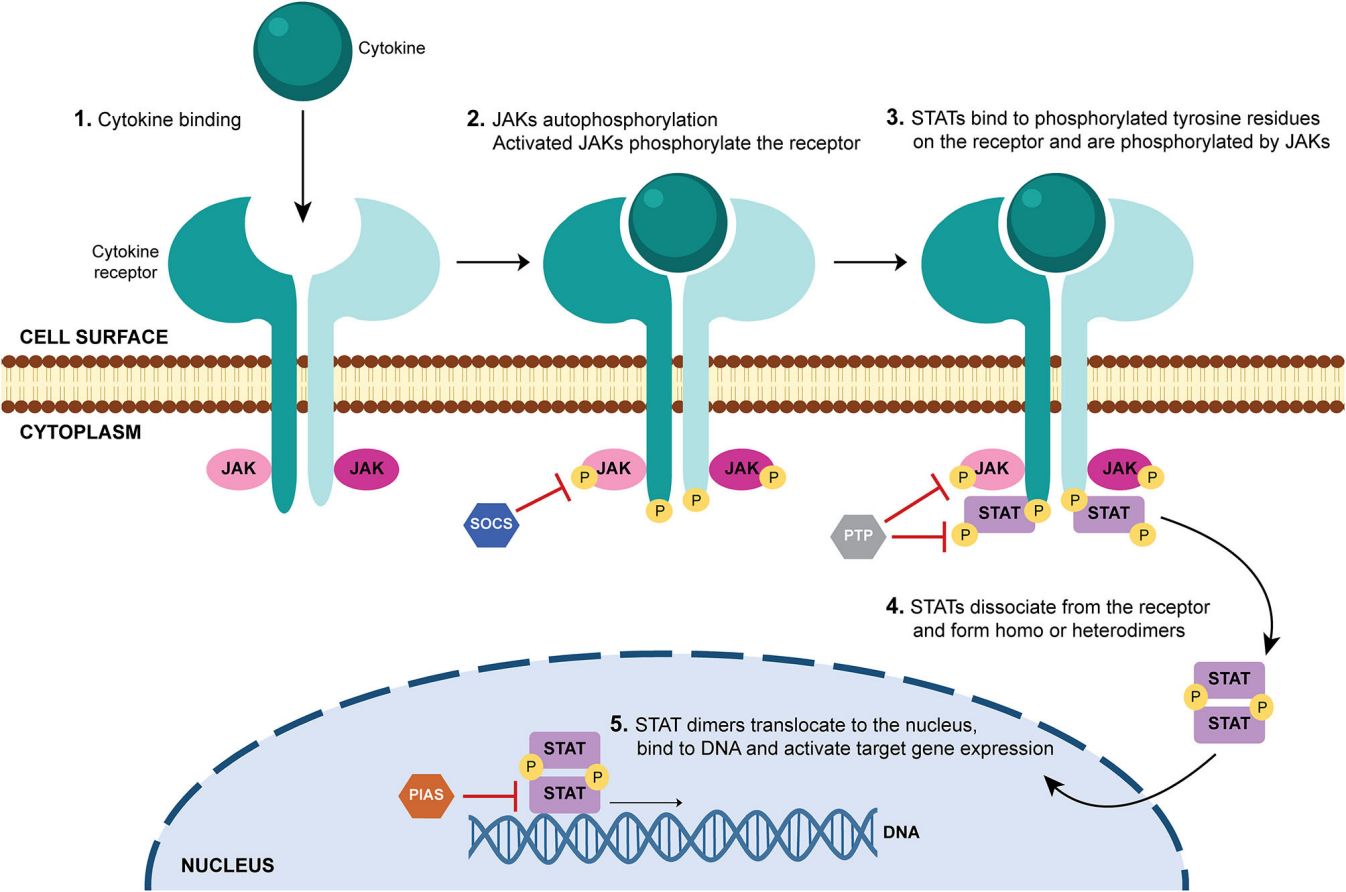
JAK-STAT signaling pathway. When a ligand (usually a cytokine) binds to its receptor in a cell, it triggers the autophosphorylation of the receptor-associated Janus kinases (JAKs). Activated JAKs phosphorylate the intracellular tail of the receptor on critical tyrosine residues, which leads to the recruitment and binding of signal transducer and activator of transcription (STAT) proteins. STATs are phosphorylated by JAKs, which induces their dissociation from the receptor. STATs form homo- or heterodimers and translocate into the cell nucleus, where they bind to specific DNA regions and activate target gene expression. Negative regulators of the JAK-STAT signaling pathway include protein tyrosine phosphatases (PTPs), which remove phosphate groups from receptors, JAKs and STATs; protein inhibitor of activated STAT (PIAS), that prevent the DNA-binding activity of STATs; and suppressor of cytokine signaling proteins (SOCS), which inhibit the activity of JAKs. DNA, deoxyribonucleic acid; JAK, Janus kinase; P, phosphate; PIAS, protein inhibitor of activated STAT; PTP, protein tyrosine phosphatase; SOCS, suppressor of cytokine signaling proteins; STAT, signal transducer and activator of transcription.

**Figure 3:**
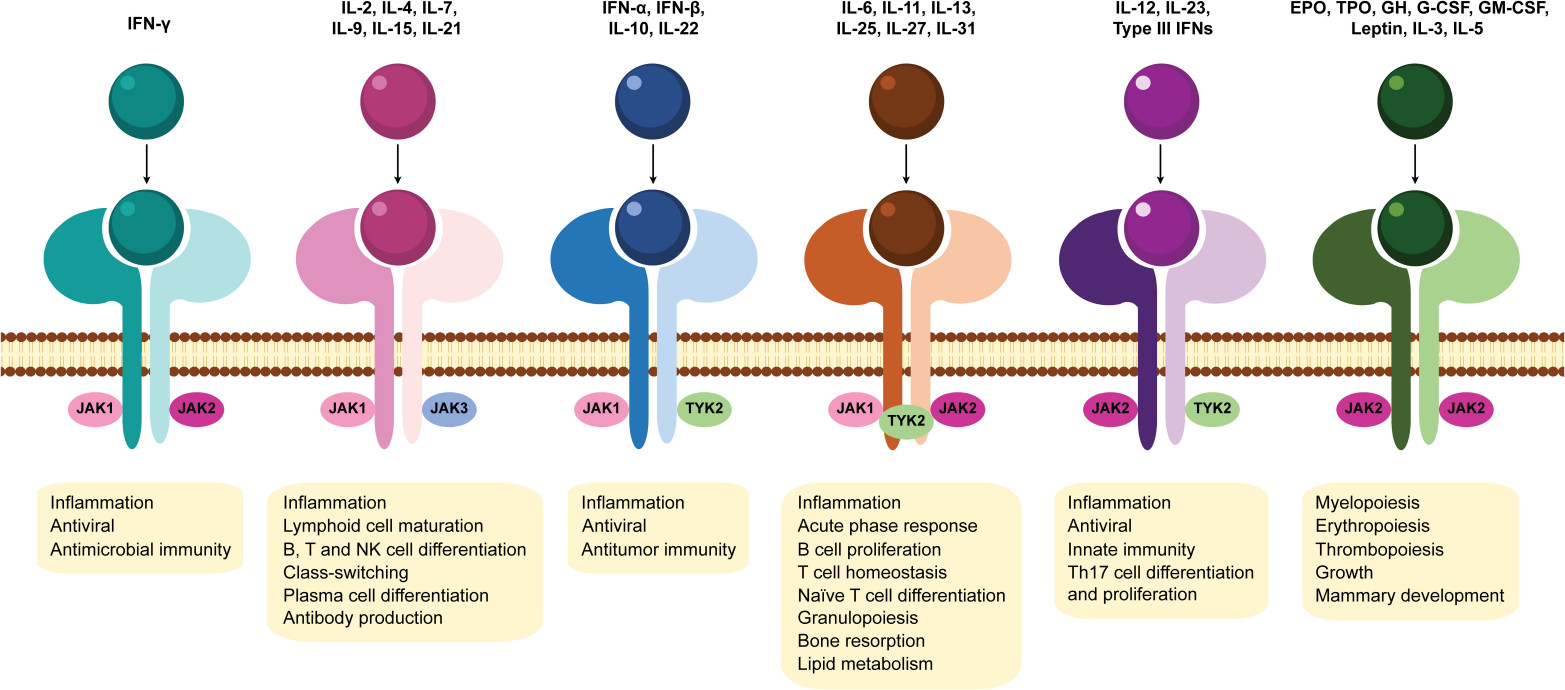
Association of Janus kinases with cytokine receptors and downstream effects of JAK-STAT signaling pathway activation. Janus kinase (JAK) family members include JAK1, JAK2, JAK3, and tyrosine kinase 2 (TYK2). Different JAK combinations with their subsequent downstream effects, each mediated by a specific subset of cytokines are represented. EPO, erythropoietin; GH, growth hormone; G-CSF, granulocyte colony-stimulating factor; GM-CSF, granulocyte-macrophage colony-stimulating factor; IFN, interferon; IL, interleukin; JAK, Janus kinase; NK, natural killer; Th17, T helper 17; TPO, thrombopoietin; TYK2, tyrosine kinase 2.

Cytokines act by binding to cell surface receptors and subsequently activate intracellular signaling cascades, such as the JAK-STAT signaling pathway. JAK-STAT signaling pathway is an evolutionarily conserved pathway that regulates many cellular processes including innate and adaptive immune responses, cell proliferation, differentiation and apoptosis. Activation of this pathway is initiated by binding of a ligand (such as interleukins, interferons, hormones and growth factors) to specific transmembrane receptors (cytokine receptors, G protein-coupled receptors, receptor tyrosine kinases and homodimeric hormone receptors) and culminates in the transcription of target genes (9, 10, 47–49) (Figure 2). JAKs, STATs and cell-surface receptors are the main key players of this signal-transduction pathway. JAKs are a family of four members of tyrosine kinases (JAK1, JAK2, JAK3, and TYK2) that selectively associate with the intracellular domains of cell receptors (50, 51) (Figure 3). JAK1, JAK2, and TYK2 are ubiquitously expressed, whereas JAK3 expression is mainly restricted to hematopoietic cells (52). Binding of a ligand to a cell surface receptor triggers the receptor dimerization and induces the autophosphorylation and activation of the receptor-associated JAKs. Activated JAKs then phosphorylate critical tyrosine residues on the receptor, which leads to recruitment of specific STATs (49, 51, 53) (Figure 2). STATs are a family of proteins named for their dual roles of transducing signals and promoting transcription of specific genes. There are seven members of the STAT family in mammals: STAT1, STAT2, STAT3, STAT4, STAT5A, STAT5B, and STAT6 (49, 54–57). After binding to the phosphorylated tyrosine residues on the receptor, STATs are phosphorylated by JAKs, which leads to their dissociation from the receptor. STATs form homo- or heterodimers and translocate into the cell nucleus via importins, where they bind to specific DNA regions and activate the transcription of target genes (Figure 2). STATs can be dephosphorylated by nuclear protein tyrosine phosphatases (N-PTPs), which leads to the inactivation of STATs. The unphosphorylated STATs associate with exportins to exit the nucleus and return to the cytoplasm where they can be reactivated for further rounds of gene transcription (10, 47, 49, 56). Overall, signaling via the JAK–STAT signaling pathway is a dynamic process that involves the rapid transmission of signal from the cell membrane to the nucleus followed by a highly organized response and subsequent controlled downregulation and attenuation of the initial signal (47–49, 54). Thus, negative regulators of the JAK-STAT signaling pathway also play an essential role. These include protein tyrosine phosphatases (PTPs), which remove phosphate groups from receptors, JAKs and STATs (58); protein inhibitor of activated STAT (PIAS), that prevent the DNA-binding activity of STATs (59, 60); and suppressor of cytokine signaling proteins (SOCS), which form a classical negative feedback loop that switches off the activity of JAKs (61, 62) (Figure 2). Disturbances in JAK-STAT signaling pathway, mostly associated with mutations (gain or loss of function) and polymorphisms in JAK and/ or STAT genes (9, 63), have been implicated in the pathogenesis of several diseases including inflammatory skin conditions (psoriasis, atopic dermatitis, alopecia areata, vitiligo) (64–71); cancers (myeloproliferative neoplasms, leukemia) (72, 73); immunodeficiencies (severe combined immune deficiency) (74); and autoimmune disorders such as RA (75–79); psoriatic arthritis (80, 81); systemic lupus erythematosus (82, 83); ankylosing spondylitis (84, 85); systemic sclerosis (86, 87); giant cell arteritis (88); sarcoidosis (89–91) and inflammatory bowel diseases (ulcerative colitis, Crohn's disease) (92, 93). Therefore, targeting JAKs and/ or STATs can be a safe and efficacious strategy for treating these diseases (94).

## JAK Inhibitors as New Treatment Options in Rheumatoid Arthritis

JAK-STAT signaling pathway has a critical role in the signal transduction of many pivotal cytokines involved in RA pathogenesis (12, 95, 96) as well as other inflammatory disorders (97). Due to their central role in the immune responses and their association with several cytokine receptors (Figure 3), the inhibition of JAKs appeared to be a promising therapeutic strategy in autoimmune diseases (94). JAK inhibitors (JAKi) represent a new class of oral drugs developed in the last decade that directly suppress the enzymatic activity of JAK family members, blocking JAK-STAT signaling pathway (12, 96). Despite the efficacy of biological DMARD treatments that target individual cytokines, biologics are large proteins that may cause immunogenicity and require either intravenous infusion or subcutaneous injection for dosing (98). In contrast, JAK inhibitors are small molecules, orally administered, that can simultaneously suppress the action of multiple cytokines. To date, five JAK inhibitors (tofacitinib, baricitinib, upadacitinib, peficitinib, and filgotinib) have been approved for the treatment of RA.

### Tofacitinib

Tofacitinib is an oral JAK inhibitor with selectivity for JAK1 and JAK3 and, to a lesser extent, JAK2 and TYK2. Tofacitinib was the first JAK inhibitor approved by the United States (US) Food and Drug Administration (FDA) (November 2012) and European Medicines Agency (EMA) (March 2017) for the treatment of moderate to severe active RA patients who had had an inadequate response or intolerance to MTX (76, 78, 99–112). Data from human clinical trial studies have demonstrated the effectiveness of the use of tofacitinib in RA patients not only as a monotherapy (at a dosage of 5 mg twice daily), but also in combination with MTX and the clinical responses have proven to be at least similar to TNF antagonists (78, 103, 105, 107, 109, 112–114). Indeed, tofacitinib has demonstrated efficacy in active RA patients by significantly improving disease activity, physical functioning, health-related quality of life as well as preventing bone erosions and structural joint damage (99, 103, 114–117). Furthermore, safety reports indicate that tofacitinib is generally well-tolerated, has a consistent safety profile (as monotherapy or combination therapy) and sustained efficacy in RA patients. However, adverse events have been described in RA patients after tofacitinib treatment with mild to moderate severity that included nausea, anemia, lymphopenia, neutropenia, lipid profile changes, increase in liver enzymes, cardiovascular events, lower respiratory tract infections, herpes zoster virus (HZV) reactivation, venous thromboembolism, and development of malignancies (76, 78, 109, 112, 114, 118–125). Nevertheless, the overall risk of infection (including serious infection) and mortality rates in RA patients treated with tofacitinib is similar to those observed in RA patients treated with biologic agents (12, 120).

### Baricitinib

Baricitinib was the second JAK inhibitor approved for clinical use in RA (in February 2017 by the EMA and in June 2018 by the FDA). Baricitinib is an oral JAK1/JAK2 inhibitor, with moderate activity against TYK2 and significantly less activity against JAK3. Approved dosages (2 and 4 mg once daily) are administered to moderate to severe active RA in adult patients who are intolerant or unresponsive to one or more DMARDs (75, 126–132). Treatment of RA patients with baricitinib monotherapy, or when baricitinib was combined with conventional synthetic DMARDs (csDMARDs) such as MTX showed efficacy and had an acceptable safety profile in early active naïve csDMARD-treated RA patients who had exhibited an inadequate response to conventional synthetic or biologic DMARDs (126, 129, 131, 132). Moreover, it has been demonstrated that baricitinib had a similar or improved efficacy when compared to TNF antagonists such as adalimumab (129, 131–134). Of note, treatment of RA patients with baricitinib was associated not only with clinical improvement, but also with inhibition of radiographic joint damage (135, 136). Overall, baricitinib is considered a safe and effective treatment in RA, although some adverse events have been described similarly to what has been observed in tofacitinib treated RA patients (132, 137–139).

### Upadacitinib

Upadacitinib is a JAK1-selective inhibitor approved by the FDA (in August 2019) and EMA (in December 2019) for the treatment of RA. Upadacitinib is indicated for the treatment of adults with moderately to severely active RA who fail to adequately respond to, or are intolerant to one or more DMARDs (77, 140–146). Upadacitinib may be used as monotherapy (15 mg or 30 mg once daily) or in combination with MTX as an effective treatment for active RA patients with an inadequate response to conventional or biological DMARDs, with an acceptable safety profile (77, 143–147). Furthermore, it has been demonstrated that upadacitinib was more effective than adalimumab treatment in ameliorating disease activity in RA patients who were concomitantly receiving MTX and significantly prevented radiographic progression (148). In addition, despite being a selective JAK1 inhibitor, upadacitinib has a similar safety profile to less-selective JAKi (139, 143, 146, 147, 149). Nevertheless, longer-term safety data are necessary.

### Peficitinib

Peficitinib is a pan-JAK inhibitor with a moderate selectivity for JAK3. It was approved for the treatment of RA in Japan in 2019 and Korea in 2020; and is currently being evaluated by the US FDA to treat adult patients with moderately to severely active RA who show inadequate response to or are intolerant of MTX (150–158). Peficitinib has been tested in RA either as monotherapy (150) or in combination with MTX (151) or csDMARDs (152) and it has been shown to significantly improve disease severity in RA patients who have an inadequate response to conventional therapies. Of note, it has been demonstrated that Peficitinib 50, 100, and 150 mg dosages administered once daily were effective in treating active RA patients, without a significant risk for adverse events (159). Overall, peficitinib has an acceptable safety and tolerability profile with similarly described adverse events as the ones reported with other JAK inhibitors (139, 153–155, 158, 160–162).

### Filgotinib

Filgotinib is a JAK1-selective inhibitor recently approved by EMA and in Japan (in September 2020) for the treatment of RA (163–170). Filgotinib is indicated for the treatment of moderate to severe active RA in adults who have responded inadequately to, or who are intolerant to one or more DMARDs. Filgotinib may be used as monotherapy (100 mg or 200 mg once daily) or in combination with MTX (168–170). Of note, similarly to upadacitinib, another selective JAK1 inhibitor, it has been demonstrated that the risks of serious adverse events did not differ between filgotinib and less-selective JAKi such as tofacitinib (168–171).

In addition to these compounds, another JAK inhibitor, decernotinib, an oral JAK3-inhibitor in Phase IIb studies (172–175), is currently under investigation for the treatment of RA. Overall, results from clinical trials with JAK inhibitors in RA are encouraging (12, 125). JAKi have shown a rapid onset of action and, in case of an adverse event, their short half-life supports a rapid reversal of immunosuppressive effects (176–178). Of note, JAK inhibitors proved efficacious when administered as monotherapy and have demonstrated a comparable or superior efficacy and safety profile to those of biologic agents (179, 180). Importantly, due to the evidence of superiority or non-inferiority of JAK inhibitors when compared to adalimumab emerging from randomized clinical trials (114, 134, 181), the 2020 updated EULAR therapeutic guidelines have recommended the use of JAK inhibitors as an alternative to biologics in RA patients refractory to cDMARDs and having poor prognostic factors, as well as in those failing a previous synthetic or biologic DMARD (182).

## Effect of JAK Inhibitors on B Cells: Evidence From the Literature

**Figure 4:**
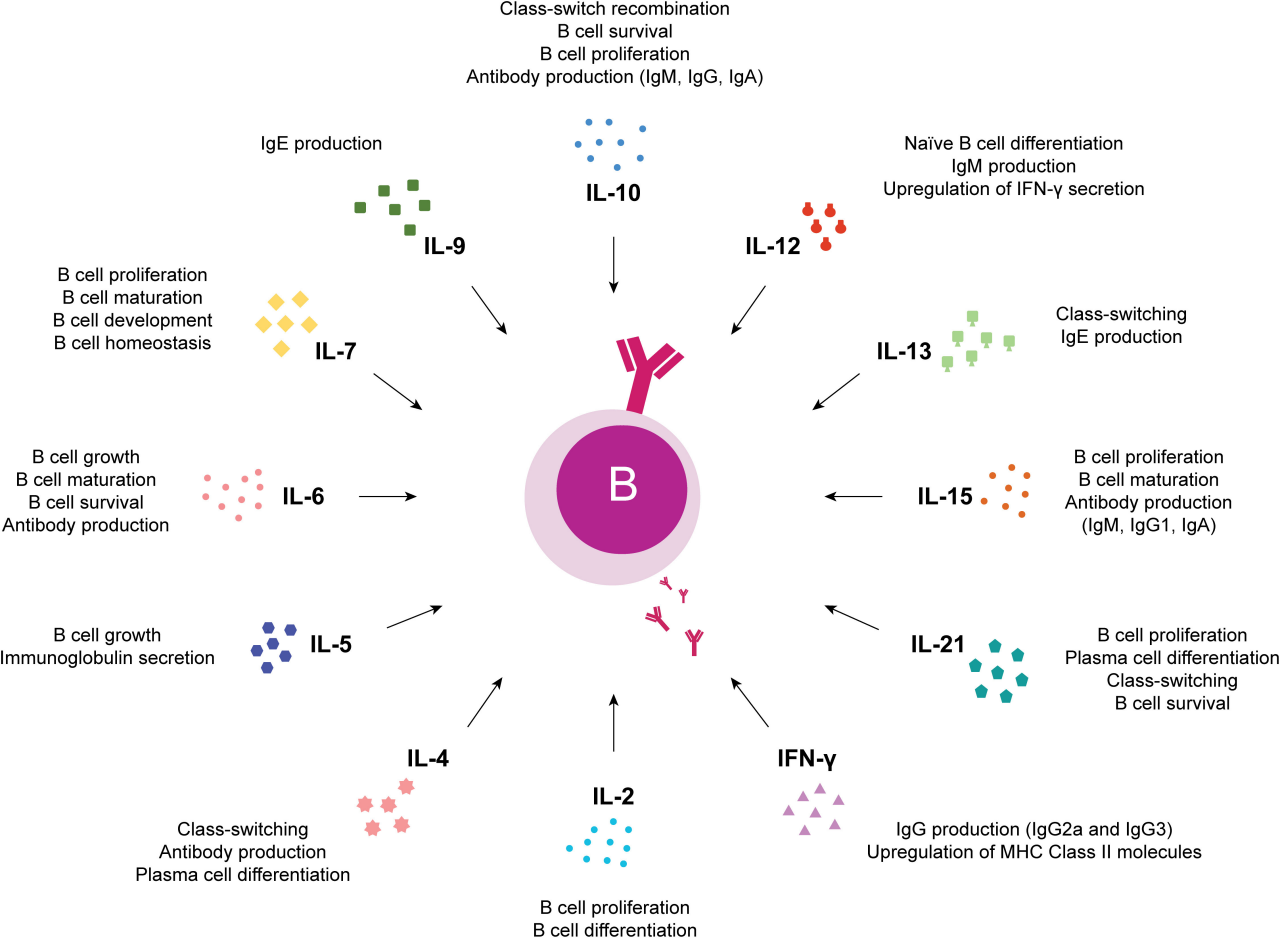
Cytokines that trigger B cell immune responses through JAK-STAT signaling pathway activation. Overview of the effects of cytokines relevant for B cells that trigger immune responses through JAK-STAT signaling pathway activation. IFN, interferon; IL, interleukin.

Studies of the effects of JAK inhibitors on circulating immune cells that play important roles in the pathogenesis of autoimmune diseases may provide insights into immunologic mechanisms associated with clinical outcomes. Due to differences in JAK targeting, JAK inhibitors may also exert distinct immunologic effects. While JAK1, JAK2, and TYK2 are ubiquitously expressed, JAK3 expression is predominantly restricted to hematopoietic cells (50, 183–186), having important roles in immune function and lymphocyte development as described in both humans (74, 187) and mice (188, 189) with JAK3 deficiencies. JAK3 mediates signaling through cytokine receptors that contain the common gamma chain (γc) or IL-2R subunit gamma (IL-2RG) including IL-2, IL-4, IL-7, IL-9, IL-15, and IL-21 receptors (51). Also, it has been shown that JAK3 is constitutively associated with CD40, an important B cell co-receptor whose signaling has a wide range of effects on B cells, including cell growth, survival, differentiation, isotype switching, rescue from apoptosis and up-regulation of expression of B7 (CD80), Fas, ICAM-1, CD23 and lymphotoxin (LT)-α (190, 191). In fact, JAK3 activating mutations are found in human hematological malignancies including B-cell lymphomas (192–194). Furthermore, observations in JAK3 knockout mice confirmed JAK3 essential role in B cell division, immunoglobulin gene rearrangement, differentiation and survival (195). Taken together, these data support that the regulation of JAK3 expression and activity is important in B cell development and function (196). Therefore, the use of JAK3 inhibitors such as tofacitinib in autoimmune diseases such as RA might have important consequences in B cell activation and function. Previous studies have shown that the primary targets of tofacitinib during pathological processes in RA are dendritic cells, CD4+ T cells such as Th1 and Th17 and activated B cells, leading to multi-cytokine targeting, decreased synovial inflammation and structural joint damage (117, 197–202). Changes in lymphocyte subsets have been documented with tofacitinib treatment (116, 176, 200, 203, 204). Indeed, phase II and phase III clinical trials involving patients with RA treated with tofacitinib showed a transient increase in total lymphocytes early in treatment, with a gradual decrease over time (204–206). In phase II RA clinical trials, variable changes in T cells were observed with short-term tofacitinib treatment, while B cells and natural killer (NK) cells increased and decreased from baseline, respectively (204, 205). Importantly, no strong association between CD4+ T cell, CD8+ T cell, B cell, or NK cell counts and serious infection incidence rates was observed (204). Although the number of studies exploring the effect of tofacitinib on B cells in the context of RA is limited, results so far indicate that tofacitinib interferes with B cell functions. In fact, it has been suggested that tofacitinib suppresses B cell activation, differentiation and class-switching, but maintains B cell regulatory function (202, 207). Moreover, tofacitinib reduces IgG and RF circulating levels in RA patients, which correlates with disease activity amelioration (200). Additionally, it was shown that tofacitinib severely impaired *in vitro* plasmablast development, immunoglobulin secretion and induction of B-cell fate determining transcription factors from naïve B cells isolated from umbilical cord blood (208). Similar, but less pronounced results were obtained with peripheral blood B cells isolated from healthy blood donors. Indeed, *in vitro* treatment of total peripheral blood B cells with tofacitinib resulted in reduced but not abolished plasmablast development, as well as reduced antibody secretion (208). Furthermore, recent studies developed in murine models of lupus have demonstrated that although tofacitinib treatment did not change B cell numbers, a significant reduction in anti-double stranded DNA (anti-dsDNA) and antinuclear antibodies (ANA) was observed in serum (209, 210). These observations pointed to the potential inability of tofacitinib-treated patients to respond to novel antigens, suggesting that vaccination against new antigens prior to tofacitinib treatment should be considered (208, 211–213). Moreover, *in vitro* activation of B cells isolated from tofacitinib treated polyarthritis patients has revealed that, in the absence of tofacitinib, B cells can be activated again and display a normal or enhanced differentiation (208). This indicates that the inhibitory effect of tofacitinib is terminated as soon as the drug is removed (176, 201, 208). Besides tofacitinib, other JAK inhibitors have been approved or are currently being tested in clinical trials as new potential treatment options for RA and/ or other autoimmune diseases and chronic inflammatory conditions. Thus, new studies concerning the effects of JAK inhibitors on innate and adaptive immune system responses are still emerging. In fact, the diversity of cytokines that trigger B cell immune responses through JAK-STAT signaling pathway activation (Figure 4) suggests that other JAK inhibitors, besides JAK3 inhibitors, might have important roles in B cell immunity (Figure 3). Changes in lymphocyte numbers (B, T, and NK cells) and subpopulations have been recently demonstrated in active RA patients after treatment with baricitinib (214). An integrated data analysis has been performed based on results from three completed phase III trials comparing placebo with baricitinib treatment (RA-BEAM, RA-BUILD, and RA-BEACON) and one ongoing long-term extension study (RA-BEYOND) in patients with active RA. Overall, a transient increase in total lymphocyte count was observed in RA patients after 4 weeks of treatment with baricitinib, returning to baseline values by week 12. Moreover, transient changes in T cells and subsets (CD3+, CD4+, CD8+, Th1, Th17, and regulatory T cells) were observed with baricitinib treatment, with cell counts remaining largely within normal reference ranges (214). Additionally, it was shown that CD19+ B cells and B cell subpopulations (including switched memory, non-switched memory, mature naïve, and immature transitional B cells) increased after 4 weeks of baricitinib treatment and remained above baseline or stabilized over time (214). Importantly, baricitinib treatment did not result in increased autoantibody (RF and ACPA) titers, suggesting that the increase in total B cell counts is unlikely to reflect a major expansion of RA antigen-specific B cells (214). Nevertheless, it is possible that some of the class-switched memory B cells, increased by baricitinib in a dose-dependent manner, are regulatory B cells, which inhibit disease progression (214). Of note, the detected changes in lymphocyte subsets were largely consistent across the baricitinib phase III RA clinical trials, which included patients with different responsiveness to prior DMARD therapies and were not associated with increased risk of serious infections (214). Recently, the *in vitro* effects of baricitinib were evaluated on human peripheral blood cells and it was shown that baricitinib modulates both innate and adaptive immune responses similarly to tofacitinib (88, 197, 215). Baricitinib suppressed the expression of costimulatory molecules (CD80/CD86) on monocyte-derived dendritic cells and inhibited T cell proliferation and differentiation of Th1 and Th17 cells. Furthermore, baricitinib suppressed the differentiation of human B cells into plasmablasts by B cell receptor and type-I interferon (IFN) stimuli and inhibited the production of IL-6 from B cells (215). Also, it was recently shown that baricitinib decreased BAFF expression in RA synovial fibroblasts similarly to tofacitinib, thus inhibiting B cell activation locally in the joints (216). The impact of baricitinib on B cells is further supported by studies developed in a mouse model of graft-vs.-host disease (GVHD) in which it was demonstrated that baricitinib inhibited the activation of allogeneic antigen presenting cells (APCs) and prevented GVHD progression (217). It was shown that baricitinib suppressed the expression of major histocompatibility complex (MHC)-II, costimulatory molecules CD80/86 and PD-L1 on B220+ and CD11c+ APCs. Moreover, baricitinib expanded regulatory T cells and downregulated Th1 and Th2 cell responses (217). Studies developed in RA patients and animal models of arthritis treated with upadacitinib have reported decreased circulating numbers of lymphocytes, neutrophils and NK cells (141, 142, 218). Nonetheless, no significant changes were detected in RF and ACPA levels in RA patients after upadacitinib treatment (144). Furthermore, it has been recently shown that upadacitinib has a generally similar profile of *in vitro* cytokine receptor inhibition observed in human leukocyte subpopulations when compared to other JAK inhibitors (219). Particularly, it was observed that upadacitinib inhibited STAT6 phosphorylation on CD19+ B cells triggered by IL-13 stimuli similarly to tofacitinib, baricitinib and filgotinib (219). However, a recent *in vitro* pharmacology study comparing tofacitinib, baricitinib and upadacitinib has revealed that different JAK inhibitors modulate distinct cytokine pathways to varying degrees (220). Notably, it was shown that upadacitinib and tofacitinib were the most potent inhibitors of the JAK1/3-dependent cytokines tested, including IL-4, IL-6 and IL-21, relevant for B cell activation, plasma cell differentiation and humoral immune responses (218, 220). In addition, studies with peficitinib have demonstrated an inhibitory effect of this JAK inhibitor on T cell activation using either a rat adjuvant-induced arthritis model (221) or human peripheral blood mononuclear cells (86, 222). Moreover, it was shown that peficitinib suppressed *in vitro* monocyte chemotactic activity and the proliferation of fibroblast-like synoviocytes from RA patients (79, 223, 224). Interestingly, decreases in neutrophil and total lymphocyte counts were observed after peficitinib treatment, but no significant changes were detected on T cell subpopulations (152–155, 158, 222, 225). Nevertheless, studies on the potential effects of peficitinib treatment on human B cells are currently lacking. Filgotinib was recently approved by EMA for the treatment of RA and clinical trials with this JAK1-selective inhibitor are currently under investigation in other autoimmune diseases. Changes in leukocyte numbers, particularly increases in B cell frequencies, have been reported in RA patients after filgotinib treatment (163, 164, 226). Furthermore, studies exploring the action of this JAKi on B cells have demonstrated that filgotinib directly inhibits human B cell differentiation and IgG production (227). Recent reports in RA patients following treatment with filgotinib have shown significant reductions in markers important for B cell chemotaxis [chemokine (C-X-C motif) ligand 13, CXCL13]; activation and survival (BAFF); regulatory function (IL-10) and germinal center and plasma cell differentiation (IL-2, IL-5, IL-7, and IL-21) (226). Moreover, filgotinib has also been shown to suppress the production of BAFF in human primary salivary gland (SG) epithelial cells and SG organoids (227). Additionally, studies developed in a mouse model of Sjögren syndrome have shown a marked reduction in lymphocytic infiltration of salivary glands after filgotinib treatment, which contributed to disease amelioration (227). Decernotinib is another JAK inhibitor currently under evaluation for the treatment of RA (173–175, 228, 229). Although lymphopenia and neutropenia have been described in decernotinib trials (174, 175), the exact mechanisms of action and effects of this JAKi on B cell immune responses still need to be further clarified. Table 1 summarizes the impact of currently approved JAK inhibitors on B cell immune responses described in the literature. Overall, additional pharmacological studies of JAKi exploring the effect of different cytokine pathways and/ or JAK targeting in distinct human leukocyte populations remain of clinical importance.

**Table 1:** Overview of the impact of JAK inhibitors on B cell immune responses based on pharmacokinetic, *in vitro* and *in vivo* studies.

**Description**	**JAK inhibitor**	**References**
Increase in B cell numbers in peripheral blood	Tofacitinib, Baricitinib, Filgotinib	(163, 164, 204, 205, 214, 226)
Suppression of B cell activation, differentiation and class-switching	Tofacitinib, Baricitinib, Filgotinib	(202, 207, 215, 216, 227)
Impairment of plasmablast development and immunoglobulin secretion	Tofacitinib, Baricitinib	(208, 215, 216)
Inhibition of antibody production	Tofacitinib, Filgotinib	(200, 208–210, 227)
Inhibition of cytokine production relevant for B cell activation and survival	Tofacitinib, Baricitinib, Upadacitinib, Filgotinib	(215, 216, 218, 220, 227)
Downregulation of the antigen presenting cell function of B cells	Baricitinib	(217)
Reduction of T helper cell responses	Baricitinib	(215, 217)
Inhibition of STAT phosphorylation on B cells	Tofacitinib, Baricitinib, Upadacitinib, Filgotinib	(219)
Downregulation of B-cell chemoattractant, activation, survival and differentiation biomarkers	Filgotinib	(226)
Decrease in B cell lymphoid infiltrates in tissues	Filgotinib	(227)

## Conclusions

JAK inhibitors are a new class of oral immunosuppressive drugs with proved efficacy in the treatment of chronic inflammatory conditions and autoimmune diseases such as RA. B cells play several important roles in RA pathogenesis since the first weeks of disease development. Pharmacokinetic, *in vitro* and *in vivo* studies developed so far with animal models of arthritis or other autoimmune conditions and/ or with human cells from RA patients or other chronic inflammatory disorders have demonstrated that JAK inhibitors (tofacitinib, baricitinib, upadacitinib, peficitinib, filgotinib and decernotinib) can affect B cell activation, proliferation and differentiation. Taking into consideration these B cell effects of JAKi and the relevant role of B cells since early RA onset it is likely that JAKi can have a major impact on the early phase of RA. Nevertheless, further research studies are necessary to clarify the exact mechanisms of action of JAKi on B cells and other immune cell targets not only in currently approved JAK inhibitors, but also in new JAKi under investigation.

## Author Contributions

RM and JF conceptualized the manuscript. RM reviewed the literature and wrote the manuscript. JF revised the manuscript and contributed with important intellectual input. All authors read and approved the final manuscript.

## Conflict of Interest

The authors declare that the research was conducted in the absence of any commercial or financial relationships that could be construed as a potential conflict of interest.
